# ML-AdVInfect: A Machine-Learning Based Adenoviral Infection Predictor

**DOI:** 10.3389/fmolb.2021.647424

**Published:** 2021-05-07

**Authors:** Onur Can Karabulut, Betül Asiye Karpuzcu, Erdem Türk, Ahmad Hassan Ibrahim, Barış Ethem Süzek

**Affiliations:** ^1^ Bioinformatics Graduate Program, Graduate School of Natural and Applied Sciences, Muğla Sıtkı Koçman University, Muğla, Turkey; ^2^ Department of Computer Engineering, Faculty of Engineering, Muğla Sıtkı Koçman University, Muğla, Turkey; ^3^ Georgetown University Medical Center, Biochemistry and Molecular and Cellular Biology, Washington, DC, United States

**Keywords:** adenovirus, host susceptibility, host-pathogen interaction, virus-host interaction, PPI prediction, viral infection prediction, virus bioinformatics

## Abstract

Adenoviruses (AdVs) constitute a diverse family with many pathogenic types that infect a broad range of hosts. Understanding the pathogenesis of adenoviral infections is not only clinically relevant but also important to elucidate the potential use of AdVs as vectors in therapeutic applications. For an adenoviral infection to occur, attachment of the viral ligand to a cellular receptor on the host organism is a prerequisite and, in this sense, it is a criterion to decide whether an adenoviral infection can potentially happen. The interaction between any virus and its corresponding host organism is a specific kind of protein-protein interaction (PPI) and several experimental techniques, including high-throughput methods are being used in exploring such interactions. As a result, there has been accumulating data on virus-host interactions including a significant portion reported at publicly available bioinformatics resources. There is not, however, a computational model to integrate and interpret the existing data to draw out concise decisions, such as whether an infection happens or not. In this study, accepting the cellular entry of AdV as a decisive parameter for infectivity, we have developed a machine learning, more precisely support vector machine (SVM), based methodology to predict whether adenoviral infection can take place in a given host. For this purpose, we used the sequence data of the known receptors of AdVs, we identified sets of adenoviral ligands and their respective host species, and eventually, we have constructed a comprehensive adenovirus–host interaction dataset. Then, we committed interaction predictions through publicly available virus-host PPI tools and constructed an AdV infection predictor model using SVM with RBF kernel, with the overall sensitivity, specificity, and AUC of 0.88 ± 0.011, 0.83 ± 0.064, and 0.86 ± 0.030, respectively. ML-AdVInfect is the first of its kind as an effective predictor to screen the infection capacity along with anticipating any cross-species shifts. We anticipate our approach led to ML-AdVInfect can be adapted in making predictions for other viral infections.

## Introduction

Adenoviruses (AdVs) are relatively large, nonenveloped, icosahedral viruses composed of a complex protein capsid surrounding the core proteins and the dsDNA genome. They belong to a diverse family called *Adenoviridae,* with several hundred recognized members capable of infecting a broad variety of cell types across several organisms (Rowe et al., 1953). As of the isolation of the first human AdV from adenoid tissues in 1953, many other novel AdVs were identified, such that, 103 human AdVs genotypes have been classified, to date, into seven “species” named A to G (Author Anonymous, 2020a). The pathogenic human AdVs (HAdV) may lead to serious gastrointestinal, respiratory, urinary, and corneal infections especially in immunosuppressed individuals (Gao et al., 2020). Moreover, recombinant AdVs are the most widely used viral vectors for gene therapy, accounting for 18.6% of vectors used in gene therapy clinical trials. AdVs feature out with their current and potential usage in different fields, including gene therapy, vaccine trials, and cancer treatments as oncolytic viruses (Singh et al., 2019).

Before any further steps leading to the infection may take place, viral pathogenesis requires the viral particle, the virion, to enter into the host cell. For AdVs, the main mechanism of entry is a two-step process, which starts with binding of a viral capsid protein (*i.e.* hexon, penton base, or mostly the fiber) to a primary receptor on the host cell to ensure attachment followed by secondary interactions to enable penetration of virion by clathrin- and dynamin-dependent endocytosis often involving integrins, or by macropinocytosis (Zhang and Bergelson, 2005; Lasswitz et al., 2018).

In explaining the pathogenesis of viral infections, therefore, understanding the viral protein–host receptor interactions plays a pivotal role. Expanding knowledge on AdV interactions, in particular, is essential not only to enhance our understanding of the life cycle, tissue tropism, host specificity/range, and cross-species transmission of the AdVs but also to help researchers in inhibiting adenoviral infections and in constructing efficient adenoviral vectors. Thus, HAdVs serve as a good template to elucidate virus–receptor interactions and as expectedly, identification and characterization of AdV receptors have been performed at varying levels of confirmation through different experimental methodologies by several investigators.

Given their diversity, broad host range, and complex use of receptors, the biological modeling of adenoviral infection poses a challenge to decipher with gaps and controversies in the existing literature. To this end, the use of computational methods on publicly available data about PPIs and the application of machine learning algorithms may accelerate and enrich our exploration of virus–host interactions. The conventional definition of PPI, however, refers to the physical contact with molecular docking between proteins that occur in a cell or in a living organism *in vivo*. As the definition implies, main databases and repositories that include PPIs are not structured from a host–(viral) pathogen point of view (De Las Rivas and Fontanillo, 2010). An exceptional resource which provides interspecies protein interaction data is the pathogen–host interaction search tool (PHISTO) (Durmus Tekir et al., 2013) which has extracted and integrated all PPIs between the human host and a non-human organism from publicly available databases and then manually labeled the respective organisms as pathogenic or not. For collected interactions without a specified method of detection, PHISTO includes a text mining module to predict the experimental method of interaction detection and also houses a user interface allowing visualization of protein networks. The recently launched pathogen–host interactions database (PHI-base), on the other hand, encompasses comprehensive expert-curated molecular and biological information, but does not cover viruses as a pathogen (Urban et al., 2020).

A similar concern also applies for the PPI prediction tools, yet there are several tools developed to predict virus–host interactions, and herein Section *Background*, we provide some background information on the publicly available virus–host PPI prediction tools DeNovo (Eid et al., 2016), HOPITOR (Basit et al., 2018), VHPPI (Alguwaizani et al., 2018), and InterSPPI-HVPPI (Yang et al., 2020) that we have used.

In the presented study, we have first curated the set of primary protein receptors that are essential in the adenoviral entry into the host cell based on the available evidence in the literature; herein Section *Adenoviral Receptors* Background, we provide further details regarding the included receptors. Then, using the public bioinformatics resources, we have identified the host species of adenoviruses, and also found the orthologs for our curated set of protein receptors in identified hosts. Similarly, we also created the set of adenoviral fiber proteins which stand for the ligands occupied in the adenoviral attachment. Next, for each of the fiber protein and adenoviral receptors, we had a dataset of pairs composed of the corresponding host and pathogen pair. Thus, altogether, we have compiled an extensive dataset on AdV–host relations. Next, we calculated the predictions as to whether there is an interaction between this particular virus fiber protein and host receptor as generated by four different existing PPI tools. Although these PPI tools are available individually, to this date, there is no approach that brings predictions of these tools together to make infection predictions. We recognize a virus-host PPI is not sufficient to warrant infection, yet attachment of the virus to a cellular receptor is a necessary condition and the initial step of viral entry which has been used previously as a decisive parameter for AdV infectivity by Hoffman et al. (Hoffmann et al., 2007; Hoffmann et al., 2008). We cannot accurately model, however, whether the viral interaction will cause its internalization or any further viral pathogenesis within the host cell. Taking these constraints into account, we used PPI as a basis for infection prediction. To this end, we applied a machine-learning, more specifically support vector machine (SVM), based methodology to develop the ML-AdVInfect predictor that uses virus-host PPI predictions from several tools in addition to the taxonomy data. This predictor is the first of its kind to carry the interaction prediction forward to anticipate whether adenoviral infection may occur in a given host species. The approach herein referred to yields a versatile and promising method to predict the occurrence of infection, investigate host-specificity, and anticipate cross-species transmissions for viral infections.

## Background

### Adenoviral Receptors

The adenoviral receptors included in the present study contains molecules that were characterized specifically as the primary, proteinaceous, surface receptor for at least one HAdV type according to the available literature, excluding glycan-based interactions, interactions with secretory proteins, as well as any other molecular interactions which are auxiliary in nature. Based on the said criteria, we curated the set of receptors composed of coxsackie and adenovirus receptor (CAR), cluster of differentiation (CD) 46, CD80 and CD86, desmoglein-2 (DSG2), integrin subunit alpha-V (ITAV), macrophage scavenger receptor 1 (MSR1), and lung macrophage scavenger receptor SR-A6 (MARCO) and a brief overview on individual receptors and experimental methodology of receptor identification is given below (Lasswitz et al., 2018; Stasiak and Stehle, 2020).

CAR is a member of the junction adhesion molecule (JAM) family within the immunoglobulin (Ig) superfamily and is present in specialized intracellular junctions. CAR functions as a receptor for all HAdV species, except for the B species and interacts with the knob domain of the viral fiber protein (Tomko et al., 1997). CD46, also known as membrane cofactor protein (MCP), is expressed on all nucleated cells and belongs to the family of regulators of complement activation. For most species B HAdVs, which do not bind CAR, CD46 was shown to function as a cellular receptor (Gaggar et al., 2003). CD80 and CD86 are expressed on the cell surface of human dendritic cells and mature B lymphocytes (Caux et al., 1994). Species B AdVs use CD80 and CD86 as receptors and the fiber knob domain is required for the interaction (Short et al., 2006). DSG2 is a protein that belongs to the cadherin superfamily and was identified as the main receptor for HAdV-3, -7, -11, and -14. Unlike CD46 interactions, high-affinity binding to DSG2 requires both penton base and fiber protein (Wang et al., 2011). Integrins are a family of transmembrane heterodimers combining into 24 proteins in vertebrates which are engaged in a plethora of cellular functions. AdVs employ various integrins via their penton protein to mainly act as co-receptors. However, in a setting with little to no CAR expression, certain integrins from the group of the αv integrins were shown to function as a primary receptor. (Lyle and McCormick, 2010; Nestić et al., 2018). Scavenger receptors constitute a large group of membrane-bound receptors. The interaction with MSR1, also designated as SR-A and CD204, was shown to be responsible for liver uptake of HAdV5 (Haisma et al., 2009). Mutational analysis of AdV capsid proteins and *in vivo* administration in mice revealed that the SR-A interaction is mediated by the hypervariable regions of the AdV hexon protein (Piccolo et al., 2013). Similarly, in murine alveolar macrophage-like MPI cells MARCO was shown to be an entry receptor for HAdV-C5 and hexon protein was suggested to be relevant to the viral ligand (Stichling et al., 2018).

The most commonly used strategies to explore any protein-protein interactions (PPIs) are yeast two-hybrid (Y2H) and affinity-purification mass spectrometry (AP-MS), in addition to other experimental modalities of array-based screening as well as flow cytometry-based binding assays, immunoadhesin/co-immunoprecipitation, luminescence, protease assays, surface plasmon resonance (SPR) and Förster Resonance Energy Transfer (FRET)-based techniques. In order to identify host factors of viral infection, initially, virus overlay protein binding assays (VOPBAs) were employed. For example, VOPBA successfully identified the AdV receptor CD46, among others (Gaggar et al., 2003) https:// www.ncbi.nlm.nih.gov/pmc/articles/PMC7094377/- bb0405. Likewise, DSG2 was confirmed as a HAdV-3 receptor through binding assays including surface plasmon resonance and gain and loss of function assay. For follow-up analysis and validation of screening hits, genetic and drug-based validation methods including CRISPR/Cas9 and RNA interference are also being utilized. Syrian hamster models have been developed as an animal model for oncolytic species C HAdV vectors; however, AdV receptor studies are otherwise based on cell culture models. From a structural biological point of view, among the primary AdV receptors, only CAR and CD46 have solved structures in complex with their adenoviral ligands according to the entries in Protein Data Bank (PDB) database (Kilcher and Mercer, 2014; Brito and Pinney, 2017; Lasswitz et al., 2018; Hensen et al., 2020; Li et al., 2020; Stasiak and Stehle, 2020).

### Machine Learning-Based PPI Prediction Tools

So far, several computational methods have been developed to predict virus-host protein interactions. As the publicly available virus-host PPI data increased, the emphasis on this subject has recently been shifted to machine-learning-based computational techniques to identify virus-host PPIs. PPI prediction tools have been developed based on different machine-learning models such as support vector machines (SVM) (Shen et al., 2007; Cui et al., 2012; Eid et al., 2016), random forest (RF) (Yang et al., 2020) and gradient boosting machine (XGBoost) (Basit et al., 2018; Chen et al., 2020).

An algorithm for predicting PPIs mediated by mimicked short linear motifs (SLiM) between HIV-1 and human has been developed by Becerra and colleagues (Becerra et al., 2017). Also, Eid and colleagues introduced an SVM-based virus-host PPI prediction model, called DeNovo, which uses amino acid sequence similarity-based features (Eid et al., 2016). Based on three PPI sets, containing several bacterial and human protein interactions, DeNovo achieved an average accuracy, sensitivity, and specificity of 97%, 94.5%, and 97.5% respectively. The most important feature that distinguishes DeNovo from other SVM-based prediction tools is that it employs a sequence similarity-based strategy for sampling the negative virus-host PPI data set for SVM training. The DeNovo sampling strategy has inspired other researchers to develop new virus-host PPI methods. HOPITOR, an XGBoost classifier-based host-pathogen predictor, is another method using the DeNovo sampling strategy. However, the sequence similarity between the different virus and host types is rather low. As a consequence, sequence similarity-based prediction methods have some limitations. To cope with this problem, Zhou and colleagues applied Naive Bayes, RF, and SVM models on feature vectors derived from amino acid compositions of interacting host-virus proteins and introduced another SVM-based tool called VirusHostPPI (Zhou et al., 2018). VirusHostPPI has been compared with two different methods, including DeNovo (Eid et al., 2016) and Barman’s SVM (Barman et al., 2014), and it achieved an accuracy of 84.47%–79.95%, the sensitivity of 80.00%–76.14% and specificity of 88.94%–83.77% against DeNovo and Barman’s SVM, respectively. As a result of the latest efforts in virus-host PPI prediction, Yang and colleagues introduced a doc2vec embedding-based RF classifier called InterSPPI-HVPPI. Using Barman et al.’s dataset, InterSPPI-HVPPI achieved 79.17% accuracy, 81.85% sensitivity, and 76.45% specificity.

In a similar manner to the overall experience in other research fields, the number of machine learning-based approaches to virus-host interaction prediction has been increasing rapidly over time, bringing a gradual decrease in the difference of performances between the developed methods. Besides, considering the host and pathogen diversity, it would be more efficient to develop new PPI prediction methods using ensemble learning techniques instead of highlighting a single method in the literature. Ensemble learning-based approaches use multiple learning algorithms to achieve greater predictive performance than is possible from any single of the constituent learning algorithms alone (Polikar, 2006; Rokach, 2010).

Here, we introduce a machine-learning-based methodology to predict AdV infections based on the utilization of an ensemble of available virus-host PPI prediction tools.

## Materials and Methods

### Identification of Adenovirus Hosts

We constructed a library of AdV hosts using the UniProt knowledgebase (UniProtKB Release 2020_02) (The UniProt, 2017), the Virus-Host DB (Mihara et al., 2016), and the National Center for Biotechnology Information GenBank (Clark et al., 2016). We initially created a list of host organisms using the curated “Virus Hosts” information available in UniProtKB for the “Adenoviridae” family, primary hosts curated in Virus-Host DB, and hosts curated in GenBank records for Adenoviridae complete genomes. Next, we parsed out the hostnames out of the AdV species names (e.g. “Human” for Human Adenovirus). The hostnames from both steps were further curated to obtain a species (or subspecies) level host organism nomenclature, reviewing the related literature and/or sequence submission records (e.g. “*Gallus gallus*” for UniProt: R4N0P7, rather than “fowl”). The list of infecting AdV species is also curated for each host and AdV–host pairs are generated.

### Creation of Adenovirus Host Receptor Protein Sets

We identified orthologs of AdV receptors in the hosts using a sequence similarity-based approach. We initially compiled the human protein sequences for the list of receptors we have manually curated, namely CAR/CXAR (UniProt Accession: P78310), CD46 (UniProt Accession: P15529), CD80 (UniProt Accession: P33681), CD86 (UniProt Accession: P42081), ITAV (UniProt Accession: P06756), DSG2 (UniProt Accession: Q14126), MSR1 (UniProt Accession: P21757), and MARCO (UniProt Accession: Q9UEW3). Human receptors are selected as a starting point, as human is the most well-studied AdV host. We ran BLAST (Altschul et al., 1990) searches with human receptor proteins (as query sequences) against locally downloaded protein sequences from UniProtKB for all the hosts with complete proteomes based on the UniProt Proteomes database. Availability of complete proteome was applied as a criterion to make sure that all orthologs are potentially represented in the respective proteomes. We parsed BLAST results to identify orthologs from various hosts using e-value and overlap thresholds. As CAR is the first-identified and most well-studied receptor in mammalian hosts, our aim was to be able to catch all the CAR orthologs in 40 host organisms in the study through BLAST searches. Moreover, we tried to avoid partial CAR orthologs or fragments. Thus, we tried different BLAST e-value and overlap thresholds, and the e-value (<1e-20) and overlap (>66%) thresholds were chosen to maximize the number of full-length orthologs of CAR receptors.

### Creation of Adenovirus Fiber Protein Sets

In order to compile a comprehensive set of AdV fiber proteins, we initially curated a fiber protein synonym list using UniProtKB adenoviridae entries to cope with naming inconsistencies. “Fiber,” “fibre,” “fiber protein,” “fiber homolog,” “protein fiber,” “fibre protein” and “fibre homolog” were among the few terms we identified as possible names assigned for the fiber protein orthologs. We then used UniProt website REST API and our terms to retrieve AdV fiber proteins. Furthermore, to account for uncharacterized fiber proteins (i.e. uncharacterized protein or hypothetical protein, or unknown), we BLAST’ed a local database of AdV sequences using the curated fiber proteins using the same e-value and overlap thresholds described in Section *Creation of Adenovirus Host Receptor Protein Sets*.

### Preparing Dataset for Adenovirus Infection Prediction

To apply machine learning classification algorithms to predict adenoviral infection, we created a dataset containing AdV–host pairs. The dataset contained all possible host and AdV pairs, where hosts are from the final list at Section *Creation of Adenovirus Host Receptor Protein Sets* and AdVs are the ones that have the fiber proteins as identified in Section *Creation of Adenovirus Fiber Protein Sets*.

For each AdV and host species pair, we computed a feature vector with two major components and a class label. The first component is predictions of virus–host protein interaction for AdV fiber protein and host receptors; the basic prerequisite for adenoviral infection. The second component is which was incorporated to account for a potential taxonomic preference toward host receptors. Finally, the class label indicates whether the AdV in question is known to infect the respective host based on the known AdV–host pairs generated in Section *Identification of Adenovirus Hosts*.

To serve as the first component of the feature vector, we utilized four virus–host PPI predictors with their respective default parameters DeNovo (run locally, using a dissimilarity threshold of 0.8), HOPITOR (run locally, with default parameters), VHPPI (online version^1^ with default parameters), and InterSPPI-HVPPI (run locally, using default specificity threshold of 0.95 as per its web site^2^). In an attempt to factor in the strengths and weaknesses of individual virus-host PPI prediction tools, and their varying prediction performance for different receptors, we applied a stacking-like ensemble technique using DeNovo, HOPITOR, VirusHostPPI, and InterSPPI-HVPPI models. For each one of 10,237 AdV–host pairs, 4 interaction predictions were computed per receptor which resulted in 32 predictions (4 predictors; DeNovo, HOPITOR, VirusHostPPI, and InterSPPI-HVPPI x 8 receptors; CAR, CD46, CD80, CD86, ITAV, DSG2, MSR1, and MARCO). Each feature in this component had a binary value; either 1 (interacting) or 0 (otherwise). For practical purposes, the lack of a specific host receptor is treated as if there were no interaction between that receptor and the fiber protein.

As the second component, we captured host taxa at four taxonomic levels; genus, family, order, and class. National Center for Biotechnology Information (NCBI) Taxonomy Database was used to gather the taxon of each organism (Federhen, 2012).

Finally, the infection class label, for each AdV–host pair, is computed to constitute the ground truth as to whether that particular AdV infects that respective host. For this purpose, we looked at the host portion in the pair to see whether it is identical to the known host of the AdV in that pair (e.g., the known host for human AdV is “homo sapiens”). If these two are identical, class label 1 is assigned as an indication of infection under the assumption that there are no cross-species transmission, while 0 is assigned as an indication of AdV being not infectious for the host in question. Consequently, the feature vectors with class label 1 (one) form the positives (i.e., adenoviral infection happens) while those with class label 0 (zero) form the negatives of our dataset.

**FIGURE 1:**
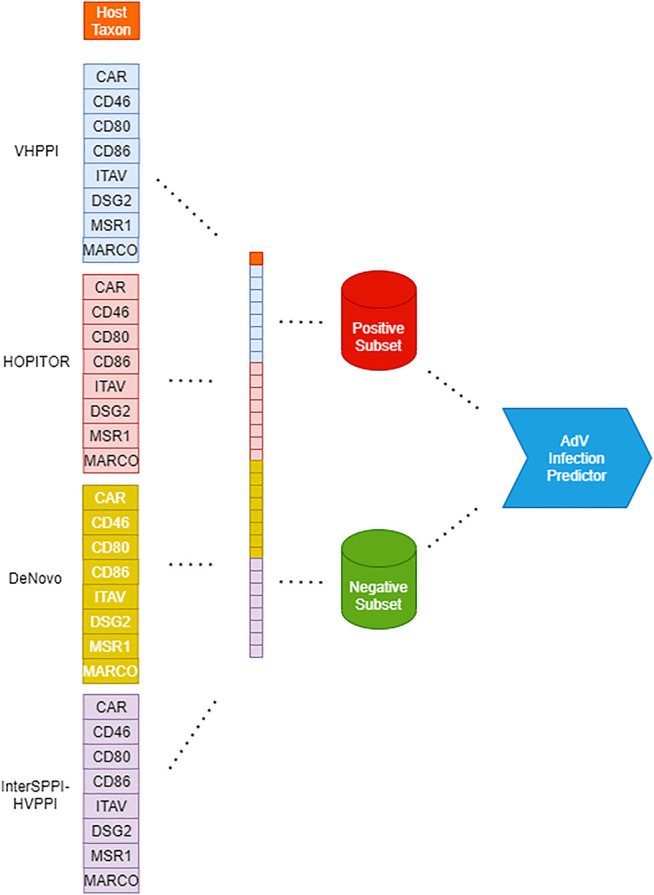
Workflow for creation of adenoviral infection prediction models from left to right: Creation of feature vector based on virus-host PPI predictors, host taxa, and infection class; partitioning of the dataset according to the class. Finally, creation of an infection predictor through various machine-learning algorithms such as RF, SVM, and MLP.

An illustration of the creation of a Dataset for Adenovirus Infection Prediction is provided as part of Figure 1.

### Creation of Adenovirus Infection Prediction Models

We used machine learning classification algorithms RF, SVM, and Multilayer Perceptron (MLP) on the dataset described in Section *Preparing Dataset for Adenovirus Infection Prediction*. The algorithms were chosen based on their use and reported performance on similar problems in bioinformatics such as virus-host protein interaction prediction (See Background). To cope with the class imbalance problem between the number of positives (i.e. adenoviral infection happens) and negatives, we employed random oversampling of minority positives set during the training of the infection prediction model. We experimented using one level of host taxa (genus, family, order, or class) at a time as part of feature vectors. For the classification algorithms requiring numerical values, host taxa which is a categorical feature are encoded using the label encoder in Scikit-Learn. For each machine learning classification algorithm, we first split our dataset into a training set (the 80% portion) to conduct hyperparameter tuning and a test set (the 20% portion) to assess respective performances. During hyperparameter tuning, we used 10-fold cross-validation where we first split the training set into 10 folds and then applied random oversampling on 9 folds which were used for training the classification model and then tested the model performance on the remaining 1 fold. It has been documented that oversampling and undersampling leads to similar performances, provided that the sampling is correctly implemented on the training folds, as we have done, during the cross-validation (Blagus and Lusa, 2015). Following the hyperparameter tuning, the best models trained on the training test (the 80% portion) are used to classify the test set for assessment of the model performances. The following performance metrics to compare our models where TP, FP, TN and FN represent the number of true positives, false positives, true negatives and false negatives, respectively. True positives (TP) contain host proteins which are predicted to correctly interact with a virus protein. True negatives (TN) are non-interactive host proteins that are correctly predicted to be non-interacting with a virus protein. False Positive (FP) is a non-interactive host protein that is wrongly predicted to interact with a virus protein. False negatives (FNs) are host proteins that are wrongly predicted to interact with a virus protein.

**Precision** measures the ability or quality of a measurement to be consistently reproduced.

$$Precision=\;\frac{TP}{TP+FP}$$

Sensitivity measures the proportion of true positives that are correctly identified.

$$\mathrm{Sensitivity=Recall}=TPR=\;\;\frac{TP}{TP+FN}$$

Specificity measures the proportion of true negatives.

$$\mathrm{Specificity}\;=\;\frac{TN}{TN+FP}$$

Accuracy is how close a measured value is to the actual (true) value.

$$\mathrm{Accuracy}\;=\;\frac{TP+TN}{TP+TN+FP+FN}$$

F-Score is a measure of a model’s accuracy on a dataset. It is used to evaluate binary classification systems, which classify examples into “positive” or “negative”.

$$F-\mathrm{Score}\;=\;\frac{2^{*}{\mathrm{Precision}}^{*}\mathrm{Recall}}{\mathrm{Precision}+\mathrm{Recall}}$$

Area Under Curve (AUC) refers to the area under the receiver operating characteristics curve which is one of the most important evaluation metrics for checking any classification model’s performance. It tells how much the model is capable of distinguishing between classes.

$$\mathrm{Area Under Curve}=\int_{a}^{b}f\left(x\right)dx$$

Matthew’s Correlation Coefficient (MCC) is used in machine learning. It is a measure of the quality of binary (two-class) classifications.

$$\mathrm{MCC}=\;\frac{TP^{*}TN-FP^{*}FN}{\sqrt{\left(TP+FP\right)\left(TP+FN\right)\left(TN+FP\right)\left(TN+FN\right)}}$$

All the machine learning models are implemented using Scikit-Learn library (2020b) and random oversampling was implemented using the imbalanced-learn toolbox (Lemaître et al., 2017) for the Python programming language. Unless otherwise is specified, the default parameters of respective implementations of RF, SVM, and MLP were used.

An illustration of the flow of our work steps from the creation of Dataset for Adenovirus Infection Prediction until the creation of Adenovirus Infection Prediction Models is provided in Figure 1.

## Results

### Adenovirus Host/Receptor and Fiber Protein Sets

**FIGURE 2:**
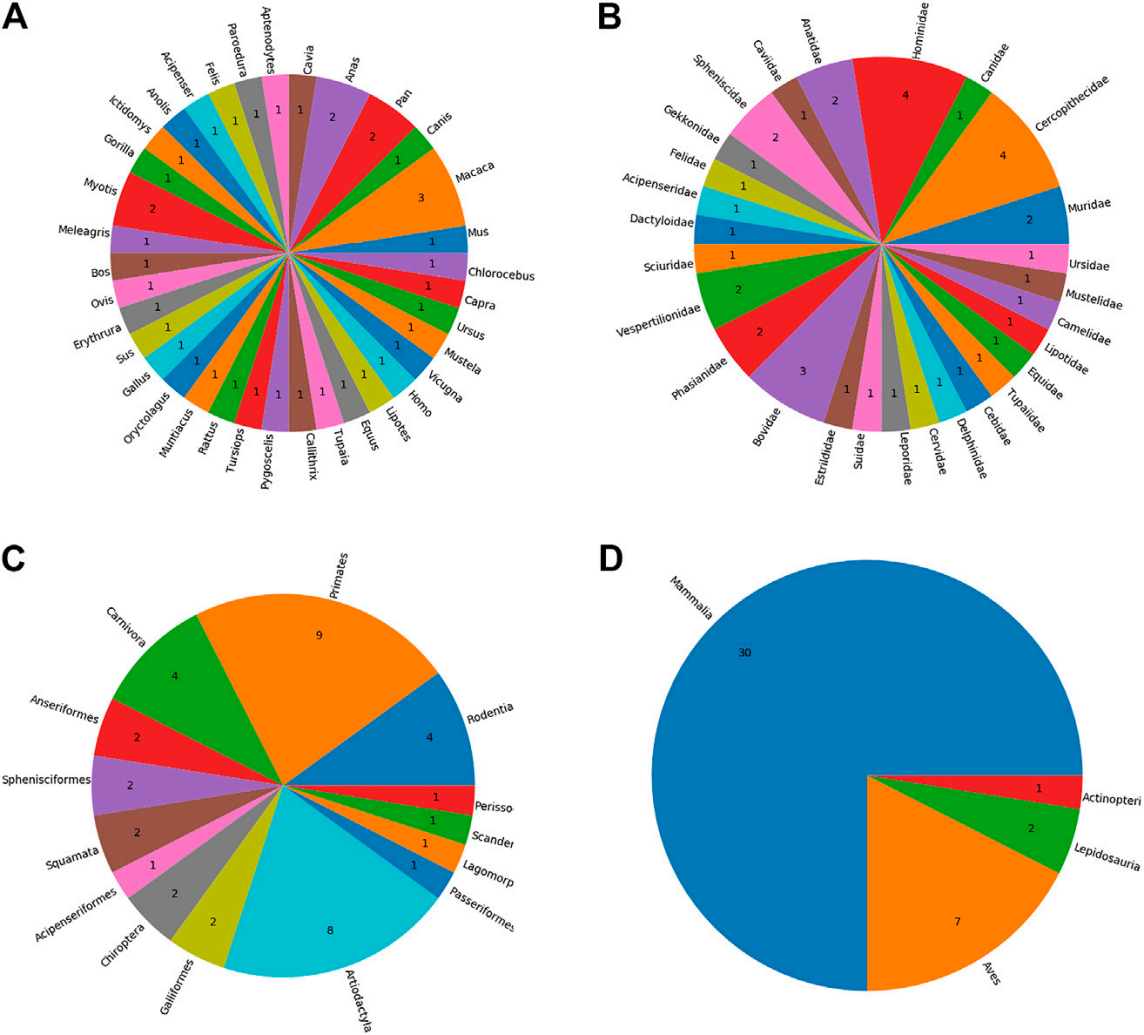
The taxonomic distribution of identified host species by **A**) genus **B**) family **C**) order, and **D**) class.

The identification of AdV hosts resulted in 297 unique species as potential host species. The majority of the hosts (n = 179) are mammalians and primates predominate this class. Once these hosts were sorted out based on the availability of the complete proteomes in UniProt, the remaining 40 host species were included in our final set of hosts. See Supplementary Table S1 for a full list of identified hosts as well as the information as to whether complete proteome data for the relevant host is available or not. Our results further confirm that the AdVs infect a wide variety of organisms including mammals, lizards, birds, turtles, and frog and toads (See Figure 2).

Out of 40 host species, CAR is found in 32 organisms, CD46 in 25, CD80 in 23, CD86 in 33, ITAV in 36, DSG2 in 38, and the scavenger receptors MSR1 and MARCO exist in 25 and 17 of these host species, respectively. For each of the 40 host species, the UniProt accession numbers for existing receptors are provided in Supplementary Table S2. As a validation, we have compared the identified receptors’ orthologs against the respective orthologs recorded in the OrthoDB database (Kriventseva et al., 2019). Our orthologs included all those recorded in OrthoDB, and further included some additional uncharacterized orthologs (eg. UniProtKB: M3Y0B3 as a CD86 ortholog in *Mustela furo*).

Our set of AdV fiber proteins is composed of 254 fiber proteins. A full list of these proteins together with the adenoviruses they belong to is provided in Supplementary Table S3.

### Dataset for Adenovirus Infection Prediction

Our dataset contains a total of 10,237 AdV–host pairs, of which 220 are from the positive class and 10,017 are from the negative class. For each AdV–host pair, each one of the 4 virus–host PPI prediction tools was used separately to make predictions for 8 host receptors. The prediction results where 1 indicates interaction and 0 indicates either no interaction or non-existence of the corresponding receptor are provided in Supplementary Table S4.

We compared the prediction results for our dataset using the correlation coefficients between individual virus–host PPI tools which are provided in Table 1. The coefficient correlations between the tools range between 0.13 and 0.79. InterSPPI-HVPPI produced a rather low number of positive predictions for the entire set of the receptors which is attributable to its conservative nature. Therefore, it has been excluded from the correlation with the other tools. The longer proteins, which are DSG-2 and ITAV (ca. 1000 amino acids) had the poorest correlation which suggests a size-dependency in the prediction of these tools.

**TABLE 1:** Correlation coefficients of PPI predictors by adenoviral receptor.

		VHPPI	HOPITOR	DeNovo			VHPPI	HOPITOR	DeNovo
CAR	VHPPI	1.00			CD46	VHPPI	1.00		
	HOPITOR	0.52	1.00			HOPITOR	0.41	1.00	
	DeNovo	0.79	0.51	1.00		DeNovo	0.55	0.54	1.00
		VHPPI	HOPITOR	DeNovo			VHPPI	HOPITOR	DeNovo
CD80	VHPPI	1.00			CD86	VHPPI	1.00		
	HOPITOR	0.14	1.00			HOPITOR	0.21	1.00	
	DeNovo	0.38	0.57	1.00		DeNovo	0.31	0.48	1.00
		VHPPI	HOPITOR	DeNovo			VHPPI	HOPITOR	DeNovo
ITAV	VHPPI	1.00			DSG2	VHPPI	1.00		
	HOPITOR	0.13	1.00			HOPITOR	0.13	1.00	
	DeNovo	0.31	0.17	1.00		DeNovo	0.52	0.23	1.00
		VHPPI	HOPITOR	DeNovo			VHPPI	HOPITOR	DeNovo
MSR1	VHPPI	1.00			MARCO	VHPPI	1.00		
	HOPITOR	0.35	1.00			HOPITOR	0.50	1.00	
	DeNovo	0.49	0.44	1.00		DeNovo	0.79	0.66	1.00

The highest correlation, on average, is between DeNovo and VHPPI followed by DeNovo and HOPITOR. The variance of correlations between the tools per receptor reinforces the use of an ensemble of virus-host PPI prediction tools rather than opting for a single one. As none of these tools correlate 100%, we anticipate each will complement each other and boost the overall performance of virus–host PPI prediction. We also validated PPI predictions against public databases. We checked PHISTO and identified that it names only human adenoviral receptor CAR and its interactions with human AdV2 and human AdV12 fiber proteins which we checked against the results from 4 PPI prediction tools and confirmed that all 4 predicted these interactions correctly.

### Comparison of Adenovirus Infection Prediction Models

We have used training set (the 80% portion) of our dataset which was generated as described in Section *Creation of Adenovirus Infection Prediction Models* for hyperparameter tuning of SVM-, RF-, and MLP-based models for adenoviral infection prediction. SVM was tested with several kernels (polynomial, radial basis function (RBF), and sigmoid) and gamma values (default = auto, 1, 10). For SVM, the highest sensitivity and AUC scores were consistently achieved with the RBF and otherwise default parameters. We experimented with MLP with activation functions ReLU and tanh along with the different hidden layer configurations. For MLP, tanh yielded the highest sensitivity and AUC scores with a hidden layer configuration of [16, 4]. RF was also experimented with several parameters including depth (50, default = 100, 150), number of trees, and split metrics (gini and entropy) where the best sensitivity and AUC scores were attained with the depth = 16, number of trees = 50, and split metrics = entropy. The results from hyperparameter tuning which were carried out without the host taxa are available in Supplementary Table S5.

The performance metrics were computed on the test split (the 20% portion) for the best SVM-, RF-, and MLP-based models which are identified through hyperparameter tuning. The 80%–20% train-test split was repeated 100 times and the mean values and standard deviation are reported in Table 2. For our study, we favored higher sensitivity models since our main focus was correctly predicting infection. The implementation of the SVM algorithm yielded the best performance in terms of sensitivity for infection prediction for our particular dataset for all the experiments (bolded in Table 2) we conducted with or without the inclusion of the host taxa levels.

**TABLE 2:** Performance metrics of adenoviral infection prediction models. RBF, Radial Basis Function; AUC, Area Under the Curve; MCC, Matthew’s Correlation Coefficient.

Host taxa level	Classifier	Sensitivity	Specificity	Accuracy	F-score	MCC	AUC
Genus	**SVM (kernel = “rbf,” gamma = “auto”)**	**0.92 ± 0.009**	**0.86 ± 0.047**	**0.92 ± 0.009**	**0.96 ± 0.005**	**0.39 ± 0.035**	**0.89 ± 0.023**
	MLP (activation = “tanh,” hidden layer=(16,4))	0.95 ± 0.009	0.73 ± 0.064	0.94 ± 0.009	0.97 ± 0.005	0.40 ± 0.045	0.84 ± 0.031
	Random forest (number of trees = 50, criterion = “entropy,” max_depth = 16)	0.96 ± 0.006	0.70 ± 0.071	0.95 ± 0.006	0.98 ± 0.003	0.42 ± 0.043	0.83 ± 0.035
Family	**SVM (kernel = “rbf,” gamma = “auto”)**	**0.91 ± 0.009**	**0.84 ± 0.057**	**0.91 ± 0.008**	**0.95 ± 0.005**	**0.36 ± 0.031**	**0.88 ± 0.027**
	MLP (activation = “tanh,” hidden layer=(16,4))	0.94 ± 0.012	0.72 ± 0.064	0.94 ± 0.011	0.97 ± 0.006	0.37 ± 0.048	0.83 ± 0.031
	Random forest (number of trees = 50, criterion = “entropy”, max_depth = 16)	0.95 ± 0.007	0.66 ± 0.068	0.95 ± 0.007	0.97 ± 0.004	0.38 ± 0.043	0.81 ± 0.033
Order	**SVM (kernel = “rbf,” gamma = “auto”)**	**0.91 ± 0.009**	**0.82 ± 0.057**	**0.90 ± 0.009**	**0.95 ± 0.005**	**0.34 ± 0.028**	**0.86 ± 0.027**
	MLP (activation = “tanh,” hidden layer=(16,4))	0.94 ± 0.011	0.70 ± 0.075	0.94 ± 0.011	0.97 ± 0.006	0.37 ± 0.051	0.82 ± 0.038
	Random forest (number of trees = 50, criterion = “entropy,” max_depth = 16)	0.95 ± 0.007	0.66 ± 0.070	0.95 ± 0.007	0.97 ± 0.004	0.37 ± 0.040	0.81 ± 0.034
Class	**SVM (kernel = “rbf,” gamma = “auto”)**	**0.88 ± 0.011**	**0.82 ± 0.061**	**0.88 ± 0.010**	**0.93 ± 0.006**	**0.30 ± 0.028**	**0.85 ± 0.029**
	MLP (activation = “tanh,” hidden layer=(16,4))	0.94 ± 0.010	0.68 ± 0.067	0.93 ± 0.010	0.97 ± 0.005	0.35 ± 0.043	0.81 ± 0.032
	Random forest (number of trees = 50, criterion = “entropy,” max_depth = 16)	0.95 ± 0.007	0.63 ± 0.071	0.94 ± 0.007	0.97 ± 0.004	0.35 ± 0.043	0.79 ± 0.035
**None**	**SVM (kernel = “rbf,” gamma = “auto”)**	**0.88 ± 0.011**	**0.83 ± 0.064**	**0.88 ± 0.010**	**0.93 ± 0.006**	**0.30 ± 0.029**	**0.86 ± 0.030**
	MLP (activation = “tanh,” hidden layer=(16,4))	0.94 ± 0.009	0.68 ± 0.079	0.93 ± 0.008	0.96 ± 0.005	0.34 ± 0.043	0.81 ± 0.038
	Random forest (number of trees = 50, criterion = “entropy,” max_depth = 16)	0.95 ± 0.007	0.63 ± 0.072	0.94 ± 0.007	0.97 ± 0.004	0.35 ± 0.041	0.79 ± 0.035

Bolded value indicates the implementation of the SVM algorithm yielded the best performance in terms of sensitivity for infection prediction for our particular dataset for all the experiments.

According to our findings, the inclusion of the host taxa level led to a slight performance improvement in terms of sensitivity, specificity and MCC. Although it was informative to see the potential benefit of inclusion of host taxa to overall predictor performance, we wanted to avoid any bias introduced by our dataset’s limited representation of the real taxonomic diversity of AdV hosts. Hence, we decided to exclude host taxa level in training models at the moment, while deferring the inclusion of host taxa to a later iteration of ML-AdVInfect when more AdV host complete proteomes become available.

For the reasons mentioned above, in this study, we chose SVM with RBF kernel model over alternative models trained without host taxa level. The analysis reported in Section *Discussion* is based on this model. Supplementary Table S4 also includes the infection predictions of this SVM with RBF kernel-based model.

In order to assess the infection prediction power of a single receptor and a single PPI prediction tool, we used the same set of machine-learning algorithms and parameters as in our hyperparameter tuning experiments described above for the overall AdV infection prediction model. The results for hyperparameter tuning for single receptor/PPI prediction tool experiments, which were carried out without the host taxa, are available in Supplementary Table S6. In turn, the performance metrics computed for the test set are available in Supplementary Table S7. Based on their performance metrics, we conclude a single-receptor-based or single-PPI-predictor-based infection prediction model is not achievable.

## Discussion

AdVs are infectious microorganisms that are particularly harmful to elderly and immunocompromised individuals. Along with their clinical importance, AdVs have further implications as they are promising vectors for gene and vaccine delivery. Therefore, adenoviral interactions with their hosts have been extensively searched. To the best of our knowledge, on the other hand, there is no computational model to estimate whether AdV can cause an infection or not in a given host. The model we propose here encompasses a machine learning-based approach to predict the infection capacity of AdVs.

In our study, we favored models trained without host taxa level as our dataset is not necessarily a representation of a wide diversity of AdV hosts. The highest sensitivity predictor among these models was based on SVM with RBF kernel with performance metrics sensitivity, specificity, and AUC 0.88 ± 0.011, 0.83 ± 0.064, and 0.86 ± 0.030, respectively. Our preference for favoring sensitivity rather than specificity is tailored toward our main goal of correctly predicting infection, but our approach does not preclude favoring higher specificity models such as MLP and RF.

In our analysis, we also identified that a single-receptor-based or single-PPI-predictor-based infection prediction model is not achievable. Yet, the overall performance of ML-AdVInfect demonstrates the utility of a stacking-like ensemble of PPI predictors for infection prediction.

In bioinformatics, several machine learning problems have to handle class-imbalanced data. Ours is not an exception to this. Oversampling techniques to randomly add instances from the minority class or undersampling techniques to randomly drop instances from the majority class are widely used on such imbalanced data (Radivojac et al., 2004; Taft et al., 2009; Kim and Choi, 2014; Li et al., 2014). Yet, as long as the cross-validation is implemented correctly, choice of sampling results in similar model performances (Radivojac et al., 2004). In the light of this, we have opted for oversampling with a correct implementation in the cross-validation process.

According to the documented results in the literature, the available virus–host PPI prediction tools (see Background) have varying performance. The level of agreement between the individual tools was limited based on our correlation analysis (coefficients at a range of 0.13–0.79). This was our main motivation behind using an ensemble of these tools for infection prediction. As our model strictly relies on the performance and use of virus–host PPI prediction tools, improvement in the performance of existing ones and/or the introduction of newly developed ones may help to attain better infection predictions.

We have addressed the main adenoviral entry mechanism into the cells, namely, binding to the primary membrane receptor on the host cell by the viral ligand (namely, CAR, CD46, CD80, CD86, ITAV, DSG2, MSR1, and MARCO) yet it is worth to emphasize that occurrence and spread of adenoviral infection may also make use of interactions between non-proteinaceous portions of molecules, viral binding to soluble host proteins, secondary interactions between the virus and host, as well as the internalization of the virion through caveolin- or clathrin-dependent mechanisms. Similarly, ligand-wize, our dataset comprises merely the AdV fiber proteins which are the most common but indeed are not necessarily the only domain of viral binding. Here, we pursued an approach to ensure the proven determinants of infection are encompassed through manual curation of a set of receptors. This approach can be expanded from both virus and host side to accommodate other interacting proteins if needed.

Although we tried to identify primary human adenoviral receptors and their orthologs to our best effort, we cannot rule out the possibility that there may still be uncharacterized proteins in various hosts or partially sequenced host genomes. Hence, we restricted our dataset to include 40 complete proteomes as curated by UniProt. As a future insight, completed proteomes might be added to this dataset as they become available.

Cross-species transmission of viruses corresponds to the capacity of a virus species to infect other host organism(s) in addition to its original host. In order to assess the capability of our predictor in detecting a potential interspecies shift, we further investigated the false positives of our best predictor, namely the SVM model with RBF kernel, as they might as well catch a cross-species transmission event. Of our false-positive results, 15% accounts for the cases where a HAdV infects another non-human primate which is a well-established zoonotic shift of AdVs (Hoppe et al., 2015). Furthermore, in 26% of the cases a primate AdV was predicted to infect another primate which could potentially be an indication of cross-species transmission. For the primates, we did a literature review and inspected Virus-Host DB. One of our false positive predictions refers to the human infection caused by a titi monkey adenovirus ECC-2011. We have identified that both *Callicebus cupreus* and *Homo sapiens* were reported as host organisms infected by this virus. According to the documented transmission (Chen et al., 2011), a novel adenovirus (TMAdV, titi monkey adenovirus) was identified in a colony of titi monkeys confined in a research center who experienced fulminant pneumonia and hepatitis leading to a devastating outcome; 23 out of 65 monkeys were infected, of whom 18 were lost. Furthermore, the researcher who was in closest contact with these monkeys also developed upper respiratory symptoms and found to be seropositive, and more concerningly, also had a clinically ill family member with no colony contact who was as well tested seropositive. Most likely, this new world monkey colony has acquired the pathogen from an unknown natural reservoir, but this outbreak implies the offending pathogen is capable of breaking the species barrier and may even cause human-to-human transmission. Although remained at a smaller scale on this particular occasion, viruses that can cross the species barrier and infect a broad primate host range may lead to larger epidemics and therefore needs closer attention. Similarly, AdVs may also be transmitted within domestic settings, across humans and domestic animals (Pauly et al., 2015). Out of false positives, 34 could be attributed to the shift of AdVs host from human to domestic animals including dog, goat, and pigs.

The work presented here, namely ML-AdVInfect, is the first of its kind in terms of allowing adenoviral infection prediction. As a step toward this predictor, we have also constructed a comprehensive dataset of AdV–host interactions which may accommodate other studies on AdVs. The proposed approach is an effective predictor to screen the infection capacity along with anticipating any cross-species shifts. It is also versatile as it allows expansion by the addition of novel virus–host PPI predictors, new host organisms, and newly identified AdV species. We anticipate such expansions will make positive contributions to the overall performance of the ML-AdVInfect. Our approach that is composed of identifying hosts, host–virus interacting protein pairs, and creating a machine-learning-based model leveraging individual virus–host PPI prediction tools, can be adapted for making predictions of infection by other viruses. As a prospective work, based on our tool ML-AdVInfect together with its further expansions and/or adaptations, a web platform with a user interface will also be provided.

## Data Availability

The original contributions presented in the study are included in the article/Supplementary Material, further inquiries can be directed to the corresponding author.

## Footnotes

1
http://165.246.44.47/VirusHostPPI/Prediction

2
http://zzdlab.com/hvppi/predict.php
